# Development of a Conceptual Framework of Health Misinformation During the COVID-19 Pandemic: Systematic Review of Reviews

**DOI:** 10.2196/62693

**Published:** 2025-11-21

**Authors:** Javier Alvarez-Galvez, Jesus Carretero-Bravo, Carolina Lagares-Franco, Begoña Ramos-Fiol, Esther Ortega-Martin

**Affiliations:** 1 Computational Social Science DataLab (CS2 DataLab) University Research Institute for Sustainable Social Development (INDESS) Universidad de Cádiz Jerez de la Frontera, Andalusia Spain; 2 Department of Statistics and Operative Research Universidad de Cádiz Cádiz, Andalusia Spain

**Keywords:** misinformation, disinformation, infodemic, social media, social networks, COVID-19, review, SARS-CoV-2, coronavirus, respiratory, infectious, scoping review, conceptual framework, management, internet, technology, technologies, disease surveillance, public health, digital health, health informatics

## Abstract

**Background:**

Despite the wide variety of studies that have focused on the recent COVID-19 infodemic, defining health mis- or disinformation remains a challenge due to the dynamic nature of the social media ecosystem and, in particular, the different terminologies from different fields of knowledge.

**Objective:**

In this work, we aim to develop a conceptual framework of health misinformation during pandemic contexts that will enable the establishment of an interoperable definition of this concept and consequently a better management of these problems in the future.

**Methods:**

We conducted a systematic review of reviews to develop a conceptual framework for health misinformation during the pandemic context as a case study.

**Results:**

This review comprises 51 reviews from which we developed a conceptual framework that integrates 6 key domains—sources, drivers, content, dissemination channels, target audiences, and health-related effects of mis- or disinformation—offering a structured approach to analyze and categorize health misinformation. These 6 domains collectively form the basis of our proposed conceptual framework.

**Conclusions:**

Our results highlight the complexity and multifaceted nature of health disinformation and underscore the need for a common language across disciplines addressing this global problem in order to use interoperable definitions and advance this evolving field of study. By offering a structured conceptual framework, we also provide a valuable foundation for interventions aimed at surveillance, public communication, and digital content moderation in future health emergencies.

## Introduction

The rapid expansion of the internet and, more recently, the widespread adoption of social media platforms have led to an unprecedented acceleration in the production and dissemination of information. While these digital ecosystems have facilitated access to knowledge, they have also fostered the proliferation of misinformation. This phenomenon has increasingly drawn the attention of researchers, particularly within the social and behavioral sciences [1,2]. Over time, interest in the effects of misinformation on human behavior and social interaction has extended beyond these disciplines to fields such as health sciences, computer science, physics, and mathematics, where scholars seek to understand the cascading effects of false or misleading information [2-5]. The COVID-19 pandemic further amplified this issue, as the global health crisis created a fertile ground for the spread of misinformation. Uncertainty, urgency, and the high demand for scientific evidence contributed to the rapid circulation of misleading claims, ranging from misconceptions about disease prevention to false treatments and conspiracy theories [6,7]. Moreover, both political figures and scientists, whether intentionally or unintentionally, played a role in amplifying misinformation, sometimes by disseminating preliminary findings without sufficient scientific scrutiny [8-10]. The increasing presence of automated agents, such as social bots and artificial intelligence (AI) tools based on deep learning and large language models, has further complicated the landscape by enabling the mass production and dissemination of misleading content [11,12].

Studies on health misinformation have emerged as a specific subfield that focuses on social and health-related topics such as vaccines, epidemics and pandemics, drugs and new tobacco products, noncommunicable diseases, eating disorders and diets, and new health treatments [2,13]. However, although the most frequent topics of debate are relatively well identified, in scientific practice, we still encounter many difficulties in delimiting the concept of health misinformation, which is ultimately affecting the advancement of research in this realm of knowledge [14]. This underscores the need for a comprehensive conceptual framework capable of integrating the multifaceted elements of misinformation—its origins, pathways, and impacts—to provide both clarity and actionable insight. In fact, we can find all kinds of definitions depending on the field and focus of the study, the analytical approach, or the type of materials analyzed (eg, internet texts, tweets, posts, and online videos, among others) [15]. On the one hand, the highly dynamic nature of social media ecosystems makes it difficult to create a stable definition that characterizes all the agents involved, the topics of conversation, the channels of misinformation, and the respective health effects [2,16]. On the other hand, there is a differential use of terminology from different fields of knowledge that makes it difficult to configure a unitary language that allows us to identify the current evidence in this field of study [17].

While the concept of health misinformation had been around since the early days of the first social media platforms [18], the COVID-19 pandemic would take this term to a new dimension never known before. During this period, health misinformation has had a clear impact on the management of the health crisis in the different countries and population groups [19,20], but it has also significantly affected the scientific field and, particularly, the way scientific evidence is generated. Indeed, the need to generate rapid evidence that would take us out of the context of the health crisis has contributed to an increase in the rate of scientific production, in some cases reducing the quality of studies [21]. With the pandemic, studies based on opportunistic samples, rapid reviews, and studies led by scientists with no previous experience in the field of public health proliferated, which, despite having an altruistic purpose, may have indirectly contributed to the increase in misinformation on health issues fundamental to the proper management of the health crisis [22-24].

Consequently, although research on health misinformation and its impact on health systems has become more prevalent, clarifying the concept continues to be difficult given the constantly changing landscape of social media and the wide array of health-related issues it involves, especially within the COVID-19 context [2]. To navigate this complexity, we adopt an inclusive term of health misinformation in this study, considering any health-related claim that is based on anecdotal evidence, lacks scientific validation, or is misleading due to the absence of empirical support [11], regardless of whether it was disseminated intentionally or unintentionally. Accordingly, our definition distinguishes between 2 primary forms: misinformation, which consists of false information shared without the intent to cause harm [25], and disinformation or malinformation, which involves the deliberate creation or manipulation of false or partially true information with the purpose of misleading or harming specific individuals, social groups, institutions, or nations [26,27].

Although efforts have been made in the existing literature to construct both conceptual frameworks and taxonomies on misinformation, for example, linked to specific topics such as vaccines [28,29] or preventive measures to combat misinformation [30-32], to the best of our knowledge, there is still no conceptual model that allows for an adequate operationalization of the concept of health misinformation. To address this research gap, the present study aims to develop a comprehensive conceptual framework for health misinformation in the context of pandemics. This tool seeks to provide an interoperable definition that integrates the key dimensions of misinformation—sources, drivers, message content, dissemination channels, audiences, and health-related effects—while addressing the interdisciplinary nature of the problem. By offering a structured approach to understanding and categorizing misinformation, this study contributes to the advancement of research in this field and provides a foundation for the development of strategies to manage and mitigate misinformation in future health crises. In particular, we selected the case of COVID-19 for several reasons: (1) the wide range of agents that have been involved in the pandemic (health professionals, academics, politicians, influencers, etc); (2) the broad impact that health misinformation has had as global health phenomenon; and (3) the usefulness of a conceptual framework that can serve as a basis for the semantic interoperability of the term in interdisciplinary scientific fields and for the management of future health emergencies. Thus, the final purpose is to lay the foundations of a taxonomy of pandemic misinformation that can be extended and adjusted to other health topics, but specifically in the context of health crises.

To carry out the present objective, a systematic review of reviews was conducted (including systematic reviews, qualitative synthesis, scoping reviews, among others), from which the fundamental dimensions that would compose our conceptual framework of health misinformation would be extracted.

## Methods

### Study Design

The present systematic review of reviews was conducted in accordance with the PRISMA (Preferred Reporting Items for Systematic Reviews and Meta-Analyses) guidelines [33].

### Search Strategy

We searched in the following databases: PubMed, Scopus, Web of Science, Ovid, and Cochrane in January 2023, based on the peak of COVID-19 misinformation literature. The search was limited to papers published before January 10, 2023. The search strategy consisted of 2 parts: 1 focused on COVID-19 and the other on misinformation using Boolean terms. The following keywords to develop the search strategy were used: (1) for COVID-19: “covid 19”, “covid-19”, “sars-cov-2 infection”, “2019 novel coronavirus disease”, “2019 novel coronavirus infection”, “2019-ncov disease”, “2019 ncov disease”, “2019-ncov diseases”, “covid-19 virus infection,” “covid 19 virus infection”, “covid-19 virus infections”, “coronavirus disease 2019”, “coronavirus disease-19”, “coronavirus disease 19”, “severe acute respiratory syndrome coronavirus 2 infection”, “sars coronavirus 2 infection”, “covid-19 virus disease”, “covid 19 virus disease”, “covid-19 virus diseases”, “disease, covid-19 virus”, “2019-ncov infection”, “2019 ncov infection”, “2019-ncov infections”, “covid19”, “covid-19 pandemic”, “covid 19 pandemic”, “covid-19 pandemics”; (2) for Misinformation: “inaccurate information”, “misleading information”, “seeking information”, “rumour”, “rumor”, “gossip”, “hoax”, “urban legend”, “urban legends”, “myth”, “fallacy”, “fallacies”, “conspiracy theories”, “conspiracy theory”, “malinformation”, “disinformation”, “misinformation”.

The search strategy was adapted to each database, and the key terms used in each database are summarized in Multimedia Appendix 1.

### Eligibility Criteria

The inclusion criteria encompassed studies addressing misinformation related to health within the pandemic context, covering topics such as COVID-19, vaccination, virus-related treatments, policies, and humorous content such as jokes and memes. Reviews published in peer-reviewed journals and written in English, Spanish, and French were considered eligible. Accepted types of literature reviews included narrative, qualitative, systematic, meta-analysis, scoping, and umbrella reviews that provided insights into information sources, messages, channels, audiences, or health outcomes.

Exclusion criteria involved nonresearch papers in scientific journals (abstracts, commentaries, opinions, editorials, letters, and books) and nonscientific content reviews, including those from social media, blogs, and newspapers. Additionally, publications that did not satisfy the inclusion criteria were excluded.

### Data Extraction

The papers found were exported to Microsoft Excel 365 (Microsoft Corp) for duplicate removal and screening. Titles and abstracts were first examined by 2 reviewers (EO-M and JC-B) independently. These reviewers selected studies based on the inclusion criteria if the title or abstract contained pandemic, health, or misinformation-related terms. Papers without an abstract went directly to full-text screening. After this screening, the results were blinded so that researchers could discuss contradictions until agreement was reached. Subsequently, the first 30% of the full-text studies were reviewed twice to establish a uniform method. In case of disagreement, this was resolved by discussion and consensus among the reviewers. In case of persistent disagreement between reviewers (EO-M and JC-B), an additional protocol involving an external reviewer was applied (JA-G). Once the method to be followed was clear and defined, 4 reviewers (BR-F, CL-F, EO-M, and JC-B) screened the full text according to the inclusion and exclusion criteria. The references of the included papers were also screened to find new studies that met the selection criteria.

### Data Synthesis

Descriptive information and information on misinformation during the COVID-19 pandemic were extracted from the selected papers in 2 tables. The first table included the title, authors, topics covered, year of publication, objectives, and a summary of the authors’ conclusions. For studies that did not include the conclusions in the abstract, a summary of the conclusions was made, taking into account the most relevant aspects. On the other hand, the second table collected information on misinformation during the COVID-19 pandemic: title, source, message, channel, audience, and result of the misinformation (Multimedia Appendix 2). To perform the analysis of the misinformation data in this second table, each of its elements was classified. For this classification, some categories have been combined because of their similarity. The mass media category, for example, covers newspapers, radio, and television. Within communication channels, certain social media platforms have also been grouped, Twitter (subsequently rebranded X; X Corp) includes Twitter, Gab (Gab AI, Inc), Weibo (Weibo Corporation), and Parler; Instagram (Meta) includes Snapchat (Snap Inc) and Pinterest; Facebook (Meta) combines Facebook and Mondo Sporco; while WhatsApp (Meta) combines WhatsApp, Telegram (Telegram Messenger Inc), and WeChat. Similarly, in terms of the consequences or impact of reduced trust, a group was formed to reduce trust in government, scientists, and medical professionals.

The development of the conceptual framework was guided by a thematic synthesis approach, involving iterative coding and integration of topics across included reviews (ie, papers mentioning misinformation sources, specific message content, drivers, channels, effects, etc). Through consensus discussions among the research team, recurring patterns were grouped into 6 dimensions that represent the underlying mechanisms and manifestations of health misinformation.

### Quality Assessment of Included Reviews

To evaluate the methodological quality of the studies included in this review, we used the QUEST (Quality Evaluation of Scientific Texts) Checklist [34], a comprehensive appraisal tool specifically designed to assess the rigor and transparency of empirical research across diverse methodological designs, including quantitative, qualitative, and mixed-methods studies. QUEST comprises 20 items grouped into 6 core domains: theoretical framework, research questions and objectives, methods, results, discussion, and additional information. Each item is scored as “yes,” “no,” or “not applicable,” enabling a structured assessment of key quality indicators such as theoretical grounding, appropriateness of study design, clarity of analytical techniques, reporting of results, discussion of limitations, and ethical considerations. This instrument was chosen for its adaptability to interdisciplinary research designs.

### Conceptual Framework

Based on the references of this review, we developed a conceptual framework. With our results, we identified the main dimensions and subdimensions addressing misinformation during the COVID-19 health crisis. All subdimensions were then ranked (assigning a corresponding score) according to the relevance within their dimension and their potential impact on the level of misinformation. We determined the relevance by considering the percentage of results within their respective dimensions. Additionally, the research team assessed the potential impact on the level of misinformation of the population, considering the benchmarks of our review. Each of the items in the conceptual framework included a score that would be agreed upon by the research team that intervened in the development of the systematic review.

The conceptual framework was developed by categorizing misinformation sources based on their profile (human expert vs nonexpert and declared bot vs nondeclared) to capture their distinct roles in the dissemination process. Within bot-generated content, a key distinction was made between declared and nondeclared bots, as previous studies have shown that declared bots maintained a neutral tone during the pandemic, whereas nondeclared bots were more likely to spread negative narratives, criticize public health measures, and promote misinformation [11,24]. Additionally, the audience dimension incorporates health literacy levels, recognizing the higher susceptibility of vulnerable groups to misinformation. This integration is supported by existing research, which has consistently demonstrated that lower levels of health literacy are strongly associated with greater exposure to and belief in health-related misinformation. By structuring the framework around these factors, this model provides a comprehensive approach to understanding the pathways through which misinformation spreads and impacts public health decision-making [35,36].

## Results

### Selection of Studies

**Figure 1:**
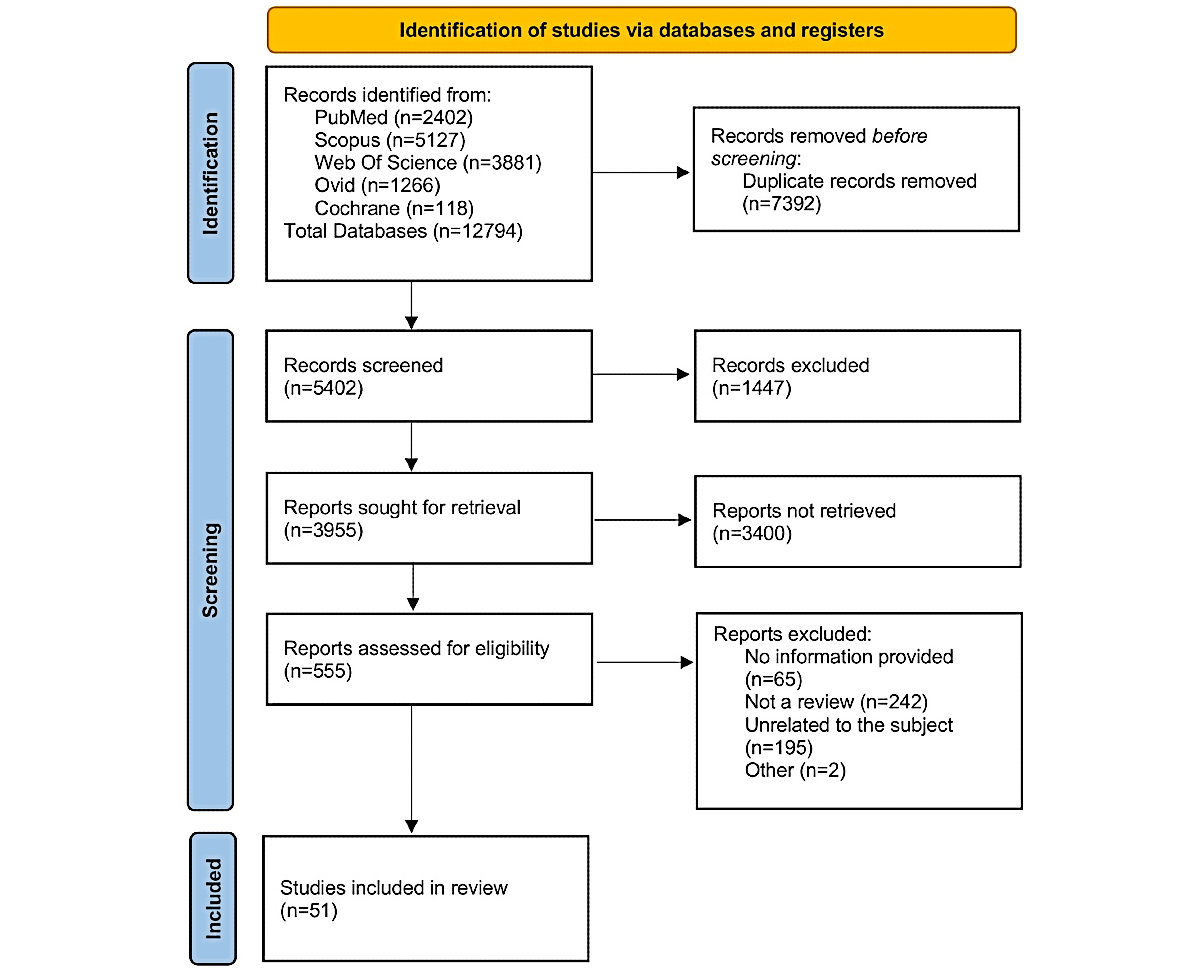
PRISMA flowchart. PRISMA: Preferred Reporting Items for Systematic Reviews and Meta-Analyses.

We identified a total of 12,794 results, from which 5402 remained after removing duplicates (Figure 1). Then, we screened titles and abstracts, selecting 556 papers for a full review. Finally, after a full-text examination, 51 papers were included in our review. Applying the QUEST instrument to our sample of included reviews, we found that most studies demonstrated high methodological quality. Of the evaluated papers, the mean number of criteria satisfied (“yes”) was 16.4 of 20, with only 2 “no” responses and 1.8 “not applicable” on average per study. Most studies provided adequate conceptual frameworks, clearly stated research questions and aims, and appropriately described their methods and analyses. However, some limitations were observed, particularly in the justification of sample sizes and the explicit reporting of data availability for replication. Detailed quality assessment results for each study are provided in Multimedia Appendix 3.

This instrument demonstrated high reliability, with reported intraclass correlation coefficients exceeding 0.98 and a Cronbach α of 0.99, indicating excellent interrater consistency and internal coherence.

**Figure 2:**
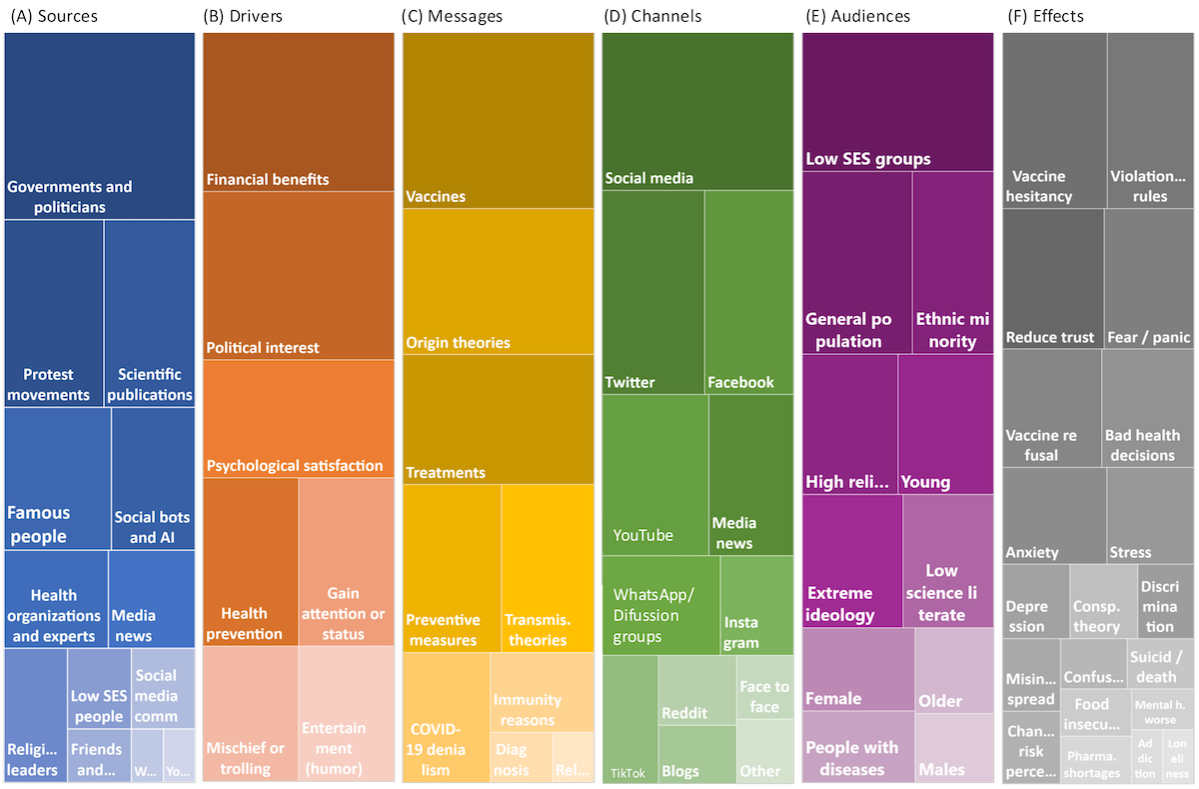
Domains and components of health misinformation during the pandemic. AI: artificial intelligence; Pharma: pharmacy; SES: socioeconomic status.

Figure 2 provides a visual depiction of the different categories that make up the 6 domains (or dimensions) that we identified as those characterizing the concept of health misinformation during the pandemic context. This group of elements is composed of (1) the different sources of misinformation, (2) the drivers, (3) the specific messages, (4) the propagation channels, (5) the various audiences, and (6) the respective social and health effects. Around this set composed of 6 dimensions, we detected a wide variability of health misinformation traits that, in this specific context, are indistinctly mentioned and combined among the 51 papers selected.

### Sources of Health Misinformation

One of the most critical aspects of health misinformation is identifying its origin. During the COVID-19 pandemic, the sources of health misinformation that were most frequently mentioned in the studies located in the literature were those related to governmental and political actors (26%) [38-59]. Among them, Donald Trump was mentioned in several papers as a figure in the spread of misinformation [44,45,49,55]. It was followed by individuals linked to protest movements (13%) [40,44-46,49,57,59-63], composed mainly of antivaxxers, and scientists (12%) [38,40,46,50,53,54,58,64-66] (including the Nobel Prize winner Luc Montagnier [67]). However, other sources were also mentioned, such as famous people (11%) [41,44,47,49,51,58,68-70], (declared and nondeclared) social bots or new AI tools (8%) [47,52,71-74], health organization experts (7%) [42,43,54,56,62,67], mass media opinion leaders (6%) [55,60,61,68,75], religious leaders (6%) [41,49-51,61], people of low socioeconomic status (4%) [41,49-51,61], and social media community members (4%) [47,55,76].

### Drivers of Health Misinformation

The studies highlight several underlying motivations behind the dissemination of health misinformation. The drivers of health misinformation during the COVID-19 pandemic were fundamentally divided between the achievement of political interest (22%) [39,41,46,49,59,63,68,71,73,76], economic interest (22%) [39,40,46,54,55,58,75-78] (eg, saying that the CDC is exaggerating the pandemic to damage Donald Trump’s reputation or selling fraudulent products, respectively [40,46,71]), and psychological satisfaction (16%) [39,47,52,58,76,79,80] or social status (11%) [41,49,68,79,80] (eg, this is the case of influencers or social media users that tried to gain attention in these new platforms [41]), which, to a certain extent, corresponded to the typology of topics mentioned as sources of misinformation in the previous section. However, misinformation was also observed as a driver of health prevention from both (scientific or health) experts, policymakers, and health organizations (11%) [39,54,61,76,81], that is, misleading, false, and erroneous information that could possibly be produced unintentionally [54,61,81]. In this sense, we found a clear division between misinformation for selfish purposes (ie, for personal or institutional gain in a context of crisis) and misinformation for altruistic purposes (ie, for helping others). Among the reasons for misinformation was also mentioned the need of troll users to damage the reputation, create chaos, directly annoy others (9%) [54,55,58,72], or entertain (9%) [54,76,79,80].

In short, while informative drivers showed a lower risk of misinformation, others, such as economic, political (which could have a greater impact on the opinions of the population), or those that were directly aimed at generating chaos and damaging the image of individuals, groups, or institutions, presented a greater risk.

### Content of Health Misinformation Messages

The health misinformation circulating during the COVID-19 pandemic covered a wide range of health-related topics. In the contents of the messages, misinformation was observed in relation to vaccines (24%) [40,41,43-47,49,51,52,54,57-63,69-72,74,76-78,80-87], where several papers related vaccines to the insertion of microchips with 5G [49,52,58,60,74,84] or the possible side effects. Furthermore, among the prevalent misinformation were various theories about the origin of COVID-19 (19%) [16,39,41,44,47,49,51,52,54,55,58-61,63,64,67,68,70,71,77-79,81,84,86], including unfounded claims suggesting that COVID-19 is a biological weapon [30,42,51,59,68,74] or is produced by the 5G network [63,68]. Additionally, we found health misinformation about certain treatments and home remedies (17%) [39-42, 44, 48, 50, 52, 54-56, 58, 61, 65, 71, 73, 75, 76, 78, 79, 81, 86-88] such as the use of herbal medicines [40,61,71,81] or even the use of cow urine [41,77]. Among these home remedies, there were also references to the use of sodium hypochlorite (ie, bleach) and alcohols to prevent COVID-19 infection [44,55,61,69,88] or even to eliminate its effects after being infected [61,77,86]. Other relevant topics were the possible preventive measures (11%) [39,41-43,49,50,54-56,58,61,69,74,76-78], theories about the transmission of the new disease (11%) [39,41-43,49,50,54-56,58,61,69,74,76-78], denialist messages about COVID-19 (8%) [44,48,49,51,52,56,59,69,73,78,87], theories about immunity (6%) [40,41,49-51,61,62,79], including the arguments about the supposed natural immunity [40,50,62] or ethnic immunity [41,49,51,61] of certain groups (ie, vegan immunity [41]), about diagnoses (2%) [41,64,76] and even with religious content (1%) [59,84], specifically about religious conspiracies, such as the false claim that the COVID-19 vaccine contained gelatin derived from pigs, leading to concerns among Muslim communities [84].

Among the “less harmful” messages, reference was made to humorous topics (satire or parody), which, although not generally intended to cause harm [54,76,79,80], had a certain potential to mislead, so that humor could also be used to intentionally spread rumors and conspiracies in a simple way. Other studies referred to false connections (when, as in the case of clickbait, headlines do not support the content), misleading content to frame information (selective selection of images, quotes, or statistics) or false contexts (when genuine content is shared with false contextual information) [39,46,58,59], practices that, as some studies point out, seem to undermine trust in the media and promote polarization. At a higher level of misinformation, studies were also located that mention of impostor content (the impersonation of genuine sources, for example, through phishing and smishing techniques), direct manipulation of content (deliberate modification of information or images with the aim of deceiving) or, in the most extreme cases, content fabrication, which would involve the creation of content that is totally false and that is created with the purpose of deceiving and causing harm (this, for example, would be the case of the so-called deepfakes) [39,58].

### Health Misinformation Dissemination Channels

A key factor in understanding health misinformation is analyzing how it spreads. Among the main channels of health misinformation, social media were frequently mentioned, which, as a whole, were identified by 80% of the studies analyzed. Many studies referred to social media platforms as the main health misinformation dissemination channel (22%) [35,38-47,49,50,52,56-58,61-63,65-68,70,71,73,74,77-79,81,86,87,89]; however, this percentage varies depending on specific platforms such as Twitter (15%) [38, 45, 47, 48, 52, 54, 55, 57, 58, 60, 65, 71-77, 79-81, 86], Facebook (13%) [40, 44, 45, 47, 50, 54-58, 60, 65, 69, 71, 74-76, 80, 81, 86], YouTube (Google LLC; 12%) [39, 40, 45, 48, 55-58, 60, 66, 69, 71, 74, 76, 78, 80, 84, 86, 89], WhatsApp and Telegram (8%) [38, 40, 45, 49, 57, 58, 60, 61, 69, 74, 76, 80, 86], Instagram (5%) [45, 56, 57, 60, 68, 74, 76, 81], TikTok (5%) [41, 45, 56, 57, 69, 74-76] and Reddit (Reddit, Inc; 4%) [38, 47, 57, 58, 74, 76]. Reference was also made to mass media news (9%) [38, 40, 42, 46, 51, 54, 57, 58, 65, 68, 70, 71, 74, 80, 86], misinformation spread through blogs (3%) [45, 54, 57, 58, 60], direct contacts in face-to-face relationships (2%) [45, 50, 57, 84], and other sources (2%) [40, 46, 55, 65], among which scientific media or web-based platforms such as LinkedIn were also mentioned [65].

In the case of channels, it was observed that much of the misinformation fell within the framework of social networks and different online platforms, while it was less common among official sources such as health organizations and the media.

### Target Audiences

Although exposure to health misinformation is transversal to different social groups, as also indicated by different studies (13% of the studies pointed to the general population [38,39,42-45,50,51,54,55,61,63,68,79,80]), a greater predisposition to misinformation was also identified among socially vulnerable population groups. Thus, for example, 20% of the studies pointed to groups with low socioeconomic status [19,39,41,43,45,46,49,51,52,55,57,59,61,62,70,73,75,76,81,82,84], ethnic minorities (10%) [39,41,50,52,61,62,73,79,81,82,84], high religiosity groups with low scientific literacy (9%) [40,43,46,49-52,62,75,84], young people because of their greater exposure to online media (9%) [39,46,49,52,55,61,75,76,80,84], extreme ideological groups (ie, commonly partisan individuals who are ideologically positioned and less critical of their own groups; 9%) [46,52,59,66,68,70,71,76,78,84], people with low scientific and health literacy (8%) [41,46,47,51,52,59,67,70,75], women (6%) [45,51,52,57,80,82,84], people with illness or disability (6%) [41,51,55,56,60,61], older adults (5%) [41,52,61,75,86], while others pointed to the greater exposure of men (4%) [52,75,76,84]. In this sense, despite the generalized susceptibility of the population to health misinformation, a higher risk was observed in groups with lower socioeconomic status (including vulnerable groups such as ethnic minorities) and religious positions (9%), as well as in those individuals who were more exposed to extreme political and lower scientific literacy (8%).

Among the various types of audiences, health literacy was observed as a fundamental articulating variable when processing and evaluating the various health contents. For this reason, to facilitate the classification in the conceptual framework, we would opt for this indicator divided into 4 categories, ranging from 0 health literacy (0 points) to high health literacy (4 points).

### Social and Health Effects of Misinformation

The impact of health misinformation extends beyond simple misconceptions, influencing both social trust and individual behaviors. The effects of health misinformation identified in the specialized literature were varied. Among the most relevant impacts were identified the increase in doubts about vaccination (13%) [40,46,47,49,51,52,57,59,62,66,70,73,76,78,80-87,89], as well as the violation of the norms established by political agents (11%) [38,41,47,50-54,63,66,68,73-76,78,80,84,85], the generalized reduction of trust in government, politicians, health institutions, and scientists (10%) [38,41,43-46,50,52,53,58,60,66,68,75,78,80,81], and the increase in vaccine refusal behavior (8%) [40,43-45,54,59,60,62,63,70,73,78,83,84]. However, as a whole, the significant effects that health misinformation may have had on the mental health of the population were also noted. The different effects included an increase in fear and panic among the population (9%) [38, 41, 42, 45, 46, 51, 52, 54, 56, 66, 71, 75, 81, 86, 89], anxiety (7%) [39,41,42,52,54,57,66,71,74,75,77,86], incorrect health decision-making [38,39,46,47,50,53,55,56,58,63,79,86,87] (8%, eg, an increase in advanced lung cancer cases among patients due to hesitancy to approach health care facilities is seen [87]), stress (6%) [42,46,52,55,56,66,71,78,81], depression (3%) [42,46,52,66,77,86], changes in risk perception (3%) [39,54,56,70,71], confusion (2%) [52,78,81,86], worsening of general mental health (2%) [66,78,86], increase in suicides (2%) [41,42,77,87], loneliness (1%) [66,81], and addictions (1%) [41,46], problems related to mental health which together accounted for 44% of the impacts identified. However, the impact of health misinformation on discrimination (3%) [41,42,46,54,71], propagation of erroneous or poor quality health information (3%) [39,55,66,68,75], conspiracy theories about the COVID-19 pandemic (3%) [47,52,58,60,68,80], impulsive buying of food and consumables (2%) [41,52,71,78], or shortage of medicines (2%) [50,55,66,78] were also mentioned.

### Conceptual Framework

**Figure 3:**
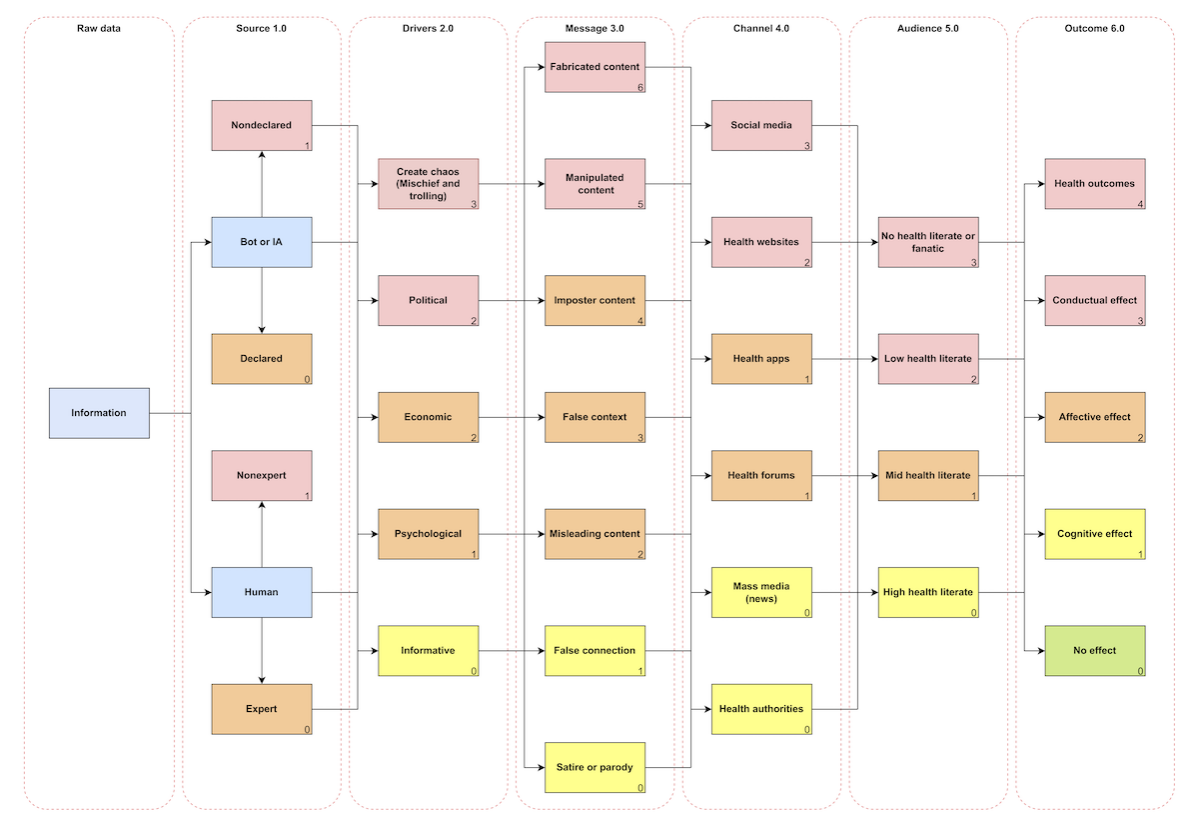
HMCF6: the conceptual framework illustrates the flow of misinformation from reception to impact. From left to right, the process begins with the reception of information, which is then classified by source type (eg, human or nonhuman and expert or nonexpert), followed by categorization of the message content (eg, misleading, fabricated, or false context). The information is then disseminated through specific channels such as social media, mass media, or health apps, and reaches different audience groups based on their level of health literacy (low, medium, or high). Finally, the process results in potential effects—cognitive, affective, or behavioral—or may produce no effect at all. This sequential model provides a structured understanding of how misinformation travels and influences public health outcomes. AI: artificial intelligence; HMCF6: health misinformation conceptual framework with six dimensions.

Figure 3 presents a conceptual framework on COVID-19 misinformation that comprehensively systematizes the 6 identified dimensions characterizing the various types of health misinformation that have emerged during the recent pandemic: (1) sources; (2) drivers; (3) types; (4) channels; (5) audiences; and (6) health misinformation effects on COVID-19. For the development of these dimensions, the results of this review were considered, as well as some references for the source and audience. First, the sources are divided into human sources [45,54,56,57,67,81] and artificial sources (social bots or AI) [46,47,52,71-73], both of which may or may not be experts. For example, in the case of artificial sources, we would distinguish between declared and nondeclared artificial sources, that is, social bots or AIs that could be declared (self-identifying as bots, usually for informational purposes) and undeclared (those acting for some covert purpose). This basic distinction would allow us to differentiate between expert and nonexpert sources, while also differentiating the positive work of social bots (eg, at the level of providing information) versus the negative effects that bots or covert AIs might generate.

In the case of drivers, we would move on a continuum ranging from offering health information with the goal of helping [39,54,61,76,81] to the extreme case of trolling [54,55,58,72] whose objective would be to create chaos (ie, damaging reputation or annoying others, sometimes without even a clear interest beyond personal satisfaction). The humorous messages [54,76,79,80] would be defined as the least harmful, assuming that audiences would be able to grasp the double meaning of the information (although logically it is not free of misunderstandings), while at the other extreme, we would have content fabrication. The channel would be divided along a gradient from information from health expert communication channels to open use of information by non–health expert groups, covering the following categories: social media [40,41,53,65,73,89], health websites, health apps, health forums, mass media [38,40,58,65], and health authorities, as the least likely to misinform. Taking into account the wide diversity of profiles of the misinformation target groups, the types of audiences would be delimited by the level of health literacy, and the effects would be as follows: no effects, cognitive effects, affective effects, behavioral effects, and, finally, direct impact on physical and mental health [38,47,63,66,71,73,78]. Table 1 provides a full operational description of the 6 dimensions.

**Table 1:** Operationalization of conceptual framework dimensions.

Dimension, application, and category				Example
**Source**				
	**Source profiling and classification to detect recurrent misinformation actors**			
		Undeclared bot or AI^a^	Automated Twitter account spreading antivaccine narratives	
		Declared bot or AI	Verified health bot disseminating WHO^b^ updates	
		Nonexpert human	Celebrity sharing unverified home remedy	
		Expert human	Physician issuing early advice without evidence	
**Drivers**				
	**Intent detection via motive-based message tagging**			
		Create chaos or trolling	Troll account spreading fake death rates to incite panic	
		Political	Post undermining public health policy during election	
		Economic	Promoting false cure to sell supplements	
		Psychological	Seeking attention via viral misinformation	
		Informative	Health worker unintentionally sharing outdated guidelines	
**Message**				
	**Risk-level classification of misinformation types based on verifiability and fabrication**			
		Fabricated content	Claim that vaccines contain surveillance chips	
		Manipulated content	Edited video misrepresenting a public health official	
		Imposter content	Fake website mimicking the CDC^c^ logo	
		False context	Old photo presented as a current event	
		Misleading content	Selective use of statistics to discredit vaccines	
		False connection	Clickbait headline about vaccine danger	
		Satire or parody	Humorous meme misinterpreted as fact	
**Channel**				
	**Channel mapping and platform-specific risk alerting**			
		Social media	Facebook groups spreading antimask claims	
		Health websites	Alternative health blog promoting nonevidence-based remedies	
		Health apps	Unregulated app suggesting treatments based on symptoms	
		Health forums	Discussion thread recommending dangerous practices	
		Mass media	Sensationalist television report linking vaccines to infertility	
		Health authorities	Official health bulletin (less likely to misinform)	
**Audience**				
	**Segmentation of target populations by health literacy or vulnerability**			
		Not health literate or fanatic	Users in conspiracy groups believing 5G causes COVID-19	
		Lowly health literate	Older adult individuals forwarding chain messages on WhatsApp	
		Mid health literate	General public with moderate science education	
		Highly health literate	Medical professionals engaging in fact-checking	
**Outcome**				
	**Monitoring behavioral, emotional, or cognitive impacts postexposure**			
		Health outcome	Delayed cancer diagnosis due to fear of hospitals	
		Behavioral effect	Refusal to wear masks or get vaccinated	
		Affective effect	Anxiety and panic from repeated exposure to false claims	
		Cognitive effect	Confusion about the safety of vaccines	
		No effect	Message ignored or dismissed due to lack of credibility	

^a^AI: artificial intelligence.

^b^WHO: World Health Organization.

^c^CDC: Centers for Disease Control and Prevention.

According to the different dimensions and categories, the resulting conceptual framework assigns numerical scores to different elements to represent their relative impact on the spread and consequences of health misinformation. These scores range from 0 to 20, where 0 indicates no impact and 20 represents the highest level of health misinformation risk. The source of misinformation is assigned a baseline score (0), acknowledging that different actors—whether human or artificial—can promote misinformation depending on their nonexpertise or undeclared character (1). Drivers of misinformation go from unintentional misinformative motives (0) to specific motives for deceiving or harming others (3). Message types are ranked on a scale from 0 to 6, with satirical or parodic content receiving the lowest score (0) due to its lower likelihood of deception, while fabricated content ranks highest (6) as it represents fully false and intentionally misleading information. Dissemination channels are evaluated between 0 and 3, with health authorities at the lower end of the spectrum (formal channels) and social media at the upper end (informal channels), highlighting their dominant role in the rapid and widespread diffusion of misinformation. The audience’s health literacy level also affects susceptibility, with individuals with high health literacy scoring the lowest (0) in comparison with fanatic audiences (3) in terms of vulnerability to misinformation. Finally, the outcomes of misinformation are structured into 4 levels, ranging from no effect (0) to severe health outcomes (4), which include cognitive, affective, and behavioral consequences that can ultimately lead to detrimental health decisions. As a rule, when a misinformation message falls into multiple categories within a given domain (eg, both mass media and social media as sources), the highest applicable score within the framework should be used. This approach ensures that the scoring system provides a consistent and quantifiable assessment of misinformation dynamics, thereby facilitating the identification of the most critical intervention points for mitigating its impact.

Table 2 presents concrete examples of health misinformation messages and their classification according to the proposed conceptual framework. It also includes an estimated risk score based on the scale defined in Figure 3, indicating their relative harmfulness across dimensions.

Based on the application of the conceptual framework to the examples in the table, the severity and potential impact of different types of health misinformation can be assessed. First, messages that combine high fabrication in content, dissemination through high-reach channels (eg, social media), and targeting of vulnerable audiences (eg, low health literacy or fanatic groups) consistently result in higher total risk scores. These examples, such as vaccine microchip conspiracies or bleach remedies, not only pose direct health risks but also undermine public trust and amplify uncertainty. Second, the source and driver dimensions are critical in distinguishing between unintentional misinformation (eg, experts presenting misinformative messages) and strategically crafted disinformation, with politically, economically, or trolling motivated cases scoring significantly higher (eg, misinformative content). Third, the framework presents content validity in differentiating between harmful and low-impact content—evidenced by the much lower score of a satirical meme, which carries minimal risk when its humorous intent is recognizable. Overall, the framework enables a nuanced evaluation that integrates both structural (source or channel) and psychosocial (audience or effect) factors, providing a robust foundation for prioritizing intervention strategies.

**Table 2:** Examples of health misinformation, framework mapping, and MRAS^a^.

Misinformation example and domain and category			Examples		Risk score
**1. “COVID-19 vaccines contain microchips for surveillance” (social bot, source: Telegram, in a low health literate group)**					
	Source	Undeclared bots		1	
	Driver	Political distrust		2	
	Content	Fabricated conspiracy		6	
	Channel	Telegram and Facebook		3	
	Audience	Low health literacy		2	
	Effect	Vaccine hesitancy		3	
	MRAS	Total		17	
**2. “Bill Gates, pharmaceutical companies, 5G, vaccines, chips... it’s all connected. They want to control us.” (singer, source: Instagram, Facebook, and Twitter)**					
	Source	Famous person (nonexpert)		1	
	Driver	Psychological		1	
	Content	Fabricated conspiracy		6	
	Channel	Instagram, Facebook, and Twitter		3	
	Audience	General public (mid health literates)		1	
	Effect	Vaccine hesitancy		3	
	MRAS	Total		15	
**3. “Hydroxychloroquine—I don’t know, it’s looking like it’s having some good results. That would be a phenomenal thing.” (politician, source: Twitter)**					
	Source	Political figures (nonexpert)		1	
	Driver	Political agenda		2	
	Content	Conspiracy theory		6	
	Channel	Mass or social media		3	
	Audience	Polarized groups (fanatic)		3	
	Effect	Polarization and distrust		3	
	MRAS	Total		18	
**4. “COVID-19 is turning people into zombies” (humorist, source: television comedy)**					
	Source	Social media users (nonexpert)		1	
	Driver	Informative		0	
	Content	Satirical misinformation		0	
	Channel	Instagram and Twitter		3	
	Audience	General public (mid health literates)		1	
	Effect	Misinterpretation		1	
	MRAS	Total		6	

^a^MRAS: Misinformation Risk Assessment Scores.

## Discussion

### Principal Findings

Our systematic review of reviews of health misinformation during the COVID-19 pandemic provides a comprehensive characterization of the sources, drivers, message content, channels, audiences, and health and social outcomes associated with the spread of misinformation. The findings presented in this paper highlight the complexity and multifaceted nature of health misinformation, emphasizing the need for the use of a common language among the various disciplines addressing this global problem to use interoperable definitions.

The prominence of government and political actors, especially the mention of Donald Trump, as major sources of misinformation underscores the polarizing capacity of political figures on public perception [90]. Additionally, the influence of protest movements, scientific publications, social bots and AI, celebrities, health organizations, and mass media evidences the great diversity of sources that can contribute to the spread of misinformation [47,59,91-94]. This highlights the need to recognize these varied origins to develop interventions aimed at different social groups (eg, by limiting access to underage groups, through fact-checking of content, or the establishment of rules against the deliberate creation and manipulation of content [37]). However, it is striking to note the confusion between misinformation sources and misinformation channels. Health studies refer to social networks as “misinformation sources,” whereas, from the perspective of communication sciences and social sciences, they could be considered “misinformation channels.” In this sense, the need for a clear conceptualization becomes evident to address the problem adequately [15], which points to the need to incorporate training programs aimed at the adequate dissemination of health information by professionals [95].

In the case of drivers, the identification of political, economic, and psychological motivations as determinants of health misinformation in the context of the recent pandemic reaffirms the interconnectedness of misinformation with broader societal issues (national elections, political debates, new markets and products, viral marketing strategies, and new ways of working and relating to others on social networking platforms). Similarly, it is worth highlighting the relevance of the unintentional production of misinformation for disease prevention and health promotion (including from the perspective of health experts). Although the ultimate goal of these forms of misinformation is usually altruistic (assuming that the aim of professionals is to help), it can also have a significant impact on opinions, attitudes, and behaviors that, directly or indirectly, can affect the health of the population when the evidence is erroneous [21,22].

The wide variety of misinformation, covering topics such as vaccines, treatments, the origin of COVID-19, preventive measures, transmission theories, denialist messages, immunity, diagnoses, and religious content, underscores the adaptability of misinformation to different dimensions of the pandemic [96]. This wide range of misinformation highlights the multifaceted nature of the infodemic [6], where false or misleading information infiltrates various aspects of public health discourse. The variety of issues uniquely linked to the pandemic demonstrates the difficulty of developing a single definition of health misinformation [15], a fact that again underscores the need for a shared conceptual framework upon which to advance the development of concrete and tailored definitions of different health issues susceptible to misinformation (vaccines, eating disorders, treatments, among others). These definitions should not only recognize the complexity of the sources and drivers of misinformation but also provide an appropriate framework for developing effective interventions to mitigate the impact of misinformation on health [2].

The significant role played by social media platforms, including Twitter, Facebook, YouTube, WhatsApp, and Telegram, in the spread of misinformation underscores the pressing need for collaborative efforts between these platforms and regulatory authorities to effectively mitigate the spread of false information. The dynamic social media ecosystem demands a nuanced understanding of the intricate relationship between traditional mass media and emerging social networking platforms [94,97]. While these platforms offer unprecedented reach and immediacy, they also present challenges related to the rapid circulation of information, the amplification of certain narratives, and the potential for the viral spread of misinformation [75]. In navigating this complex landscape, it is crucial to recognize that social media platforms operate as influential channels for information consumption, shaping public perceptions and influencing attitudes on a global scale [88]. As observed in this study, efforts to address misinformation should not only focus on these platforms in isolation but also consider the broader media ecosystem, where mass media news plays a role alongside social media, and direct interpersonal communication. In this context, a holistic approach to addressing health misinformation involves collaboration between social media platforms, traditional media outlets, health authorities, and other stakeholders [94,98]. This collaboration is essential for developing comprehensive strategies that encompass content moderation, fact-checking mechanisms, and educational campaigns aimed at promoting media literacy [94]. Understanding the synergies and interplay between mass media and social media is fundamental for creating effective interventions that can withstand the challenges posed by the rapid dissemination of information in the digital age [99]. Therefore, an integral and collaborative approach is necessary to build resilience against the detrimental effects of misinformation on public perception and, ultimately, public health.

Health misinformation affects the entire population, but it is crucial to recognize specific vulnerable groups, such as those with low incomes, ethnic minorities, and people with low scientific literacy. These groups face unique challenges due to socioeconomic disparities, cultural differences, and unequal access to quality education and scientific knowledge. Therefore, addressing misinformation in these groups requires adapted strategies considering the intersectionality of their social profiles [35]. For example, people with low socioeconomic status may encounter barriers to accessing accurate information, ethnic minorities may face cultural misunderstandings, or even more educated people may sometimes assume certain information arrogantly and uncritically. Thus, understanding the differential susceptibility among these population groups is critical to designing targeted interventions and, ultimately, building their resilience against misinformation.

Additionally, the diverse and wide-ranging effects of health misinformation on trust in institutions, vaccination rates, adherence to (sometimes misguided) treatments, adherence to health norms, and mental health highlight the need to address this problem from a global perspective. The substantial effects on mental health outcomes, including fear, stress, anxiety, and depression (especially in young groups), emphasize the urgent need for mental health support services in future infodemics.

Given the growing risk of future infodemics, our proposed conceptual framework offers not only a systematized understanding of health misinformation but also a foundation for developing actionable tools. It can inform the design of real-time surveillance systems that monitor emerging misinformation by categorizing it according to its source, content, or dissemination channel. Similarly, it provides valuable guidance for crafting targeted public health messaging aimed at vulnerable audiences identified as being at greater risk. The framework also has applications in enhancing content moderation systems by enabling the detection of high-impact misinformation patterns—for instance, fabricated claims spread by undeclared bots directed at individuals with low health literacy. By classifying misinformation across its key dimensions, more precise and effective countermeasures can be implemented to limit its reach and mitigate its consequences. These practical applications underscore the framework’s relevance for navigating the complex and evolving landscape of digital misinformation. Despite increased scholarly interest, few studies have contextualized the problem with such specificity. Existing models tend to focus on isolated aspects or topics, whereas our framework introduces an integrated and operational structure applicable to both academic analysis and intervention strategies [99-101]. Therefore, our framework enables systematic classification across all 6 domains, facilitating tailored responses such as educational initiatives, content moderation, or targeted fact-checking.

Our study contributes to the current debate on misinformation by offering a specific health-oriented conceptual framework, contrasting with broader approaches focused on science-related misinformation [100,101]. While the academic consensus has identified the multiple misinformation sources, dissemination mechanisms, and impacts of misinformation on science, our work refines this understanding by specifically addressing the health crisis context and, in particular, the impact of health misinformation on attitudinal and health outcomes at the individual level. While previous studies have developed taxonomies and conceptual frameworks on misinformation, often focusing on general classifications, media dissemination, or the risks associated with medical communication and social media, this work proposes a more integrative approach by providing a structured framework specifically tailored to health misinformation, which underscores the necessity of a common language and semantic interoperability. Therefore, this conceptual framework has the potential to enhance the management of current and future health emergencies (ie, epidemics, pandemics, natural disasters, and humanitarian crises) by facilitating the rapid identification and classification of health misinformation dimensions within the evolving digital communication landscape.

Beyond identifying the 6 individual dimensions, our findings suggest important interrelations among these elements that shape the dynamics of health misinformation. For instance, certain sources (such as political figures or nondeclared bots) often align with specific drivers such as political or economic agendas, thereby amplifying misleading content through high-reach channels such as social media. Similarly, vulnerable audiences characterized by low health literacy may be disproportionately exposed to complex misinformation narratives, intensifying affective and behavioral health impacts. These interconnected pathways underscore that the spread and impact of health misinformation are not merely additive but synergistic, with drivers and channels interacting to reinforce certain message types and ultimately influence trust, perceptions, and health behaviors. Recognizing these interdependencies provides a more holistic understanding of how misinformation ecosystems function, which is essential for designing targeted, multidimensional interventions.

### Limitations and Strengths

This study has several limitations. First, given the rapid advancement of technology, recent publications on bots, AI, or large language models may have emerged after the completion of our review. Second, while misinformation studies have historical roots dating back to the early 20th century, their interaction with evolving technologies and digital health communication is a more recent phenomenon. The expansion of online platforms and social media has significantly altered the drivers and mechanisms of misinformation spread, adding layers of complexity to its study. Additionally, a language bias is present, as only publications in English, Spanish, and French were included. This may have limited the global scope of our findings, potentially excluding valuable perspectives from studies published in other languages, which could provide deeper insights into the spread of health misinformation across diverse cultural and geopolitical contexts. Moreover, for future studies, there is a need to work on the validation of the conceptual framework that has been developed from evidence in other cultural and linguistic contexts.

Despite these limitations, this study also presents several notable strengths. While technological advancements and linguistic constraints may have influenced the scope of our review, our work lays the groundwork for a taxonomy that, although initially developed in the context of the COVID-19 pandemic, can be adapted for future pandemic preparedness. Moreover, the synthesis of findings from this review underscores the urgent need for interoperable definitions of health misinformation for improved measurement of this diffused phenomenon. Given the complexity and evolving nature of misinformation, definitions should be flexible yet aligned with a common framework, facilitating comparisons across different dimensions, including sources, drivers, content, channels, audiences, and impacts. This study highlights that addressing health misinformation and its negative effects requires a multilateral approach involving researchers, governments, social media platforms, health organizations, and communities, as well as the necessity for targeted interventions and educational campaigns tailored to specific demographic groups.

### Conclusions

This study highlights the complexity and multifaceted nature of health misinformation during the COVID-19 pandemic, emphasizing the need for an interoperable conceptual framework to facilitate the identification, measurement, and management of this socially harmful phenomenon. By characterizing the main sources, drivers, message types, dissemination channels, audiences, and health-related effects of misinformation, our findings underscore the importance of a shared language across disciplines to improve understanding and intervention strategies. The development of a structured approach to health misinformation contributes not only to advancing research in this field but also to enhancing public health responses in future crises. Furthermore, the impact of misinformation on vulnerable groups, trust in (health) institutions, and overall public health outcomes underscores the necessity of targeted interventions that integrate media literacy, fact-checking mechanisms, and collaborative efforts between researchers, policymakers, health organizations, and digital platforms. As the digital information ecosystem continues to evolve, addressing misinformation requires a dynamic and interdisciplinary approach that accounts for both technological advancements and societal behavioral patterns. Our proposed framework serves as a foundational step toward mitigating the spread of health misinformation and fostering a more resilient and informed society in the face of future health crises.

## Appendix

Multimedia Appendix 1 Search strategy.

Multimedia Appendix 2 Data collection.

Multimedia Appendix 3 PRISMA checklist.

## Abbreviations

AI: artificial intelligence
PRISMA: Preferred Reporting Items for Systematic Reviews and Meta-Analyses
QUEST: Quality Evaluation of Scientific Texts
