# Supplementary material for: Development of a Conceptual Framework of Health Misinformation During the COVID-19 Pandemic: Systematic Review of Reviews
Source: JMIR Public Health Surveill. 2025 Nov 21;11:e62693. doi: 10.2196/62693 (PMC12680940; doi:10.2196/62693)
Supplement: Multimedia Appendix 1 [file publichealth_v11i1e62693_app1.docx]

**A scoping review to develop an interoperable taxonomy of health misinformation during the COVID-19 pandemic**

| **PubMed**  (“inaccurate information"[Title/Abstract] OR "misleading information"[Title/Abstract] OR "seeking information"[Title/Abstract] OR rumour*[Title/Abstract] OR rumor*[Title/Abstract] OR gossip[Title/Abstract] OR hoax*[Title/Abstract] OR "urban legend"[Title/Abstract] OR "urban legends"[Title/Abstract] OR myth*[Title/Abstract] OR fallacy[Title/Abstract] OR fallacies[Title/Abstract] OR "conspiracy theories"[Title/Abstract] OR "conspiracy theory"[Title/Abstract] OR malinformation[Title/Abstract] OR disinformation[Title/Abstract] OR misinformation[Title/Abstract]) AND (( “covid 19” [Title/Abstract] OR covid-19 [Title/Abstract] OR “sars-cov-2 infection”[Title/Abstract] OR “sars-cov-2 infection”[Title/Abstract] OR “sars-cov-2 infections” [Title/Abstract] OR “2019 novel coronavirus disease” [Title/Abstract] OR “2019 novel coronavirus infection” [Title/Abstract] OR “2019-ncov disease” [Title/Abstract] OR “2019 ncov disease” [Title/Abstract] OR “2019-ncov diseases”[Title/Abstract] OR “covid-19 virus infection”[Title/Abstract] OR “covid 19 virus infection”[Title/Abstract] OR “covid-19 virus infections”[Title/Abstract] OR “coronavirus disease 2019”[Title/Abstract] OR “coronavirus disease-19” [Title/Abstract] OR “coronavirus disease 19” [Title/Abstract] OR “severe acute respiratory syndrome coronavirus 2 infection” [Title/Abstract] OR “sars coronavirus 2 infection” [Title/Abstract] OR “covid-19 virus disease”[Title/Abstract] OR “covid 19 virus disease” [Title/Abstract] OR “covid-19 virus diseases”[Title/Abstract] OR “disease, covid-19 virus”[Title/Abstract] OR “2019-ncov infection”[Title/Abstract] OR “2019 ncov infection” [Title/Abstract] OR “2019-ncov infections”[Title/Abstract] OR covid19 [Title/Abstract] OR “covid-19 pandemic”[Title/Abstract] OR “covid 19 pandemic”[Title/Abstract] OR “covid-19 pandemics” ) )  **Filters:** english, french and spanish | 2,421 results  **With filters:**  english, french and spanish languages  2,402 results |
| --- | --- |
| **SCOPUS**  ( TITLE-ABS-KEY ( "inaccurate information" ) OR TITLE-ABS-KEY ( "misleading information" ) OR TITLE-ABS-KEY ( "seeking information" ) OR TITLE-ABS-KEY ( rumour* ) OR TITLE-ABS-KEY ( rumor* ) OR TITLE-ABS-KEY ( gossip* ) OR TITLE-ABS-KEY ( hoax* ) OR TITLE-ABS-KEY ( "urban legend" ) OR TITLE-ABS-KEY ( "urban legends" ) OR TITLE-ABS-KEY ( myth* ) OR TITLE-ABS-KEY ( fallacy ) OR TITLE-ABS-KEY ( fallacies ) OR TITLE-ABS-KEY ( "conspiracy theories" ) OR TITLE-ABS-KEY ( "conspiracy theory" ) OR TITLE-ABS-KEY ( malinformation ) OR TITLE-ABS-KEY ( disinformation ) OR TITLE-ABS-KEY ( misinformation ) ) AND ( TITLE-ABS-KEY ( "covid 19" ) OR TITLE-ABS-KEY ( covid-19 ) OR TITLE-ABS-KEY ( "sars-cov-2 infection" ) OR TITLE-ABS-KEY ( "sars-cov-2 infection" ) OR TITLE-ABS-KEY ( "sars-cov-2 infections" ) OR TITLE-ABS-KEY ( "2019 novel coronavirus disease" ) OR TITLE-ABS-KEY ( "2019 novel coronavirus infection" ) OR TITLE-ABS-KEY ( "2019-ncov disease" ) OR TITLE-ABS-KEY ( "2019 ncov disease" ) OR TITLE-ABS-KEY ( "2019-ncov diseases" ) OR TITLE-ABS-KEY ( "covid-19 virus infection" ) OR TITLE-ABS-KEY ( "covid 19 virus infection" ) OR TITLE-ABS-KEY ( "covid-19 virus infections" ) OR TITLE-ABS-KEY ( "coronavirus disease 2019" ) OR TITLE-ABS-KEY ( "coronavirus disease-19" ) OR TITLE-ABS-KEY ( "coronavirus disease 19" ) OR TITLE-ABS-KEY ( "severe acute respiratory syndrome coronavirus 2 infection" ) OR TITLE-ABS-KEY ( "sars coronavirus 2 infection" ) OR TITLE-ABS-KEY ( "covid-19 virus disease" ) OR TITLE-ABS-KEY ( "covid 19 virus disease" ) OR TITLE-ABS-KEY ( "covid-19 virus diseases" ) OR TITLE-ABS-KEY ( "disease, covid-19 virus" ) OR TITLE-ABS-KEY ( "2019-ncov infection" ) OR TITLE-ABS-KEY ( "2019 ncov infection" ) OR TITLE-ABS-KEY ( "2019-ncov infections" ) OR TITLE-ABS-KEY ( covid19 ) OR TITLE-ABS-KEY ( "covid-19 pandemic" ) OR TITLE-ABS-KEY ( "covid 19 pandemic" ) OR TITLE-ABS-KEY ( "covid-19 pandemics" ) )  **Filters:** english, french and spanish | 5,255 results  **With filters:**  english, french and spanish languages  5,127 results |
| **Web of Science**  ((((((((((((((((TS=("inaccurate information")) OR TS=("misleading information" )) OR TS=("seeking information")) OR TS=( rumour* )) OR TS=(gossip*)) OR TS=(hoax* )) OR TS=("urban legend")) OR TS=("urban legends")) OR TS=(myth*)) OR TS=(fallacy )) OR TS=(fallacies)) OR TS=("conspiracy theories")) OR TS=("conspiracy theory")) OR TS=(malinformation )) OR TS=(disinformation)) OR TS=(misinformation)) AND ((TS= ( "covid 19" ) OR TS= ( covid-19 ) OR TS= ( "sars-cov-2 infection" ) OR TS= ( "sars-cov-2 infection" ) OR TS= ( "sars-cov-2 infections" ) OR TS= ( "2019 novel coronavirus disease" ) OR TS= ( "2019 novel coronavirus infection" ) OR TS= ( "2019-ncov disease" ) OR TS= ( "2019 ncov disease" ) OR TS= ( "2019-ncov diseases" ) OR TS= ( "covid-19 virus infection" ) OR TS= ( "covid 19 virus infection" ) OR TS= ( "covid-19 virus infections" ) OR TS= ( "coronavirus disease 2019" ) OR TS= ( "coronavirus disease-19" ) OR TS= ( "coronavirus disease 19" ) OR TS= ( "severe acute respiratory syndrome coronavirus 2 infection" ) OR TS= ( "sars coronavirus 2 infection" ) OR TS= ( "covid-19 virus disease" ) OR TS= ( "covid 19 virus disease" ) OR TS= ( "covid-19 virus diseases" ) OR TS= ( "disease, covid-19 virus" ) OR TS= ( "2019-ncov infection" ) OR TS= ( "2019 ncov infection" ) OR TS= ( "2019-ncov infections" ) OR TS= ( covid19 ) OR TS= ( "covid-19 pandemic" ) OR TS= ( "covid 19 pandemic" ) OR TS= ( "covid-19 pandemics" ) ))  **Filters:** english, french and spanish | 4,046 results  **With filters:**  english, french and spanish languages  3,881 results |
| **Ovid**  ((("inaccurate information") OR ("misleading information" ) OR ("seeking information") OR ( rumour* ) OR (gossip*) OR (hoax* ) OR ("urban legend") OR ("urban legends") OR (myth*) OR (fallacy ) OR (fallacies) OR ("conspiracy theories") OR ("conspiracy theory") OR (malinformation ) OR (disinformation) OR (misinformation) ) AND ( ( "covid 19" ) OR ( covid-19 ) OR ( "sars-cov-2 infection" ) OR ( "sars-cov-2 infection" ) OR ( "sars-cov-2 infections" ) OR ( "2019 novel coronavirus disease" ) OR ( "2019 novel coronavirus infection" ) OR ( "2019-ncov disease" ) OR ( "2019 ncov disease" ) OR ( "2019-ncov diseases" ) OR ( "covid-19 virus infection" ) OR ( "covid 19 virus infection" ) OR ( "covid-19 virus infections" ) OR ( "coronavirus disease 2019" ) OR ( "coronavirus disease-19" ) OR ( "coronavirus disease 19" ) OR ( "severe acute respiratory syndrome coronavirus 2 infection" ) OR ( "sars coronavirus 2 infection" ) OR ( "covid-19 virus disease" ) OR ( "covid 19 virus disease" ) OR ( "covid-19 virus diseases" ) OR ( "disease, covid-19 virus" ) OR ( "2019-ncov infection" ) OR ( "2019 ncov infection" ) OR ( "2019-ncov infections" ) OR ( covid19 ) OR ( "covid-19 pandemic" ) OR ( "covid 19 pandemic" ) OR ( "covid-19 pandemics" ))).ti,ab,kf.  **Filters:** english, french and spanish | 1,281 results  **With filters:**  english, french and spanish languages  1,266 results |
| **Cochrane**  Search term by term | **With filters:** Published between 2019 and 2023  118 results |
| Total to screen  Without duplicate records | 12,794 results  5,402 results |
